# Outcome after acute myocardial infarction: a comparison of patients seen by cardiologists and general physicians

**DOI:** 10.1186/1471-2261-4-14

**Published:** 2004-08-06

**Authors:** Ibrahim Abubakar, David Kanka, Barbara Arch, Jo Porter, Peter Weissberg

**Affiliations:** 1 School of Medicine, Health Policy and Practice, University of East Anglia, Norwich, NR4 7TJ UK; 2 Health Protection Agency Regional Epidemiology Unit (East of England), Institute of Public Health, Cambridge CB2 2SR, UK; 3 Director of Public Health, South Cambridgeshire Primary Care Trust, Heron Court, Cambridge, UK; 4 CAMS, Institute of Public Health, Cambridge, UK; 5 Department of Cardiology, Peterborough District Hospital, Peterborough, UK; 6 Department of Cardiology, Addenbrooke's Hospital, Cambridge, UK

## Abstract

**Background:**

The management of acute myocardial infarction (AMI) has improved over the last 50 years with the more frequent use of effective medicines and procedures. The clinical benefit of the speciality of the attending physician is less clear. The United Kingdom National Service Framework for coronary heart disease (CHD) suggested that patients with CHD are likely to benefit from cardiological supervision. We set out to assess the effect of access to cardiologists on survival among AMI patients admitted in two UK hospitals.

**Methods:**

The study was conducted in a university hospital and a district general hospital in England. Information was obtained on age, sex, ethnicity, Carstairs socioeconomic deprivation category derived from postcode of residence, comorbidity, distance from hospital and medication from all patients admitted with acute myocardial infarction in two acute trusts between July 1999 and June 2000. Record linkage to subsequent Hospital Episode Statistics and Registrar General's death records provided follow up information on procedures and mortality up to eighteen months after admission. Cox proportional hazard models were used to investigate the main hypothesis controlling for confounding. The main outcome measure was 18-month survival after myocardial infarction.

**Results:**

Access to a cardiologist was univariately associated with improved survival (hazard ratio 0.16, 95% CI 0.10 to 0.25). This effect remained after controlling for the effect of patient characteristics (hazard ratio 0.22, 95% CI 0.14 to 0.25). The effect disappeared after controlling for access to effective medication (hazard ratio 0.70, 95% CI 0.33 to 1.46).

**Conclusions:**

Access to a cardiologist is associated with better survival compared to no access to a cardiologist among a cohort of patients already admitted with AMI. This effect is mainly due to the more frequent use of effective medicines by the group referred to cardiologists. Hospitals may improve survival by improving access to effective medicines and by coordinating care between cardiologists and general physicians.

## Background

The management of acute myocardial infarction (AMI) has improved over the last 50 years with the more frequent use of effective medicines and procedures. The clinical benefit of the speciality of the attending physician is less clear. The effect of the speciality of the attending physician on mortality in AMI has been studied mainly in the United States (US) with conflicting results arising from the studies [3-6]. The explanations considered for the difference in mortality observed in some of the studies include patient's condition (case mix and comorbidity)[3], volume of workload[5,7] and treatment given[3]. Previous studies show that knowledge and use of effective medicines is better among cardiologists compared with general physicians in the US [8-10]. Co-ordination of care between cardiologists and non-cardiologists also improves survival of patients seen by non-cardiologists [11].

The National Service Framework (NSF) for coronary heart disease (CHD) published in March 2000, suggested that patients with CHD are likely to benefit from cardiological supervision. To provide this level of care, all acute hospitals will eventually need a minimum of two cardiologists [1]. A recent survey by the Royal College of Physicians found an average of 1.7 whole time equivalent (WTE) cardiologists in the 211 hospitals receiving patients with AMI in the UK [2]. Eight hospitals did not have a cardiologist.

There is a need to establish whether the involvement of a cardiologist in the management of AMI patients affects the quality and outcome of care, and if so to identify ways to improve the outcome of care for patients unable to gain access to a cardiologist. This study aims to assess the effect of access to cardiologists on survival among AMI patients accounting for access to effective investigation, medication, procedures and the underlying condition of the patient at presentation.

## Methods

A retrospective cohort design was used.

### Study population and inclusion criteria

All patients admitted to two hospitals in Eastern England between 1^st ^July 1999 and 31^st ^June 2000 with a discharge diagnosis of AMI were included in the analysis. A diagnosis of AMI was based on evidence of raised cardiac enzymes and/or other indicators of myocardial necrosis and on a physician's judgement of ECG changes indicative of AMI.

### Data collection and analysis

Hospital Episode Statistics were used to extract records of all patients admitted with the ICD10 diagnostic codes for Acute Myocardial Infarction (I21, I22, I23) in any diagnostic field. The records were restructured to produce one record per patient using the new NHS number. For records with missing NHS number, a unique identifier was created using date of birth, sex and post code. The unique identifier was also used as a check for the records with NHS numbers. Records of all Finished Consultant Episodes with OPCS 4 codes of either Coronary Artery Bypass Grafting (K40–46) or Percutaneous Transluminal Coronary Angiography (K49–50) were extracted. The process was repeated for angiography (K63 – 65). Record linkage with subsequent Hospital Episode Statistics and Registrar General's records provided follow up information on procedures and death up to eighteen months after the index AMI.

All cases were reviewed by examining the case notes. Detailed information on variables summarised below was obtained.

List of variables extracted from patients' case notes

• Hospital where patient was first admitted

• Whether the patient was seen by a cardiologist or not, determined by evidence of a cardiology review in a patients case note

• The average distance from residence to hospital first seen was determined by mapping the distance in miles using the RAC™ website distance calculator

• A history of comorbidity – defined as the presence of diabetes, metastatic malignancy, cerebrovascular accident (CVA), asthma, chronic obstructive airway disease, renal impairment, endocrine disorder, chronic infection, dementia

• Physical impairment with poor mobility

• Procedures – exercise testing, angiography, CABG, PTCA

• Appropriate medication – β blockers, aspirin, ACE inhibitors, thrombolysis (table 2)

**Table 1:** Patient characteristics, and whether seen by cardiologist or by general physician

Patient characteristic	Physician	
	
	Cardiologist (N = 275)	Non Cardiologist (N = 201)
Mean age (SD)	64.4 (SD 12.8)	74.8 (SD 11.4)
Sex n (%) Male	275 (71.4)	201 (52.8)
History of:		
*Congestive heart failure %*	11.3	29.6
*Angina %*	94.9	86.7
*Hypertension %*	41.8	46.6
*Diabetes mellitus %*	12.1	17.1
*Impaired mobility %*	7.8	17.7
*Smoking %*	36.0	49.6
Impaired left ventricular function %	24.6	25.0

**Table 2:** Odds ratios for drug and procedure use after AMI by physician (cardiologist relative to non-cardiologist)

Intervention	Number (Percent)		Odds ratio (95% CI)
		
	Cardiologist (275)	Non Cardiologist (201)	
Thrombolytic therapy*	140 (51%)	66 (33%)	2.08 (1.35 – 3.23)
β blocker on discharge*	206 (75%)	90 (45%)	3.70 (2.38 – 5.56)
Aspirin*	264 (96%)	160 (80%)	6.67 (3.23 – 14.29)
ACE inhibitors	124 (45%)	80 (40%)	1.27 (0.01 – 1.92)
Exercise testing	192 (70%)	46 (23%)	7.70 (4.76 – 12.50)
Coronary angiography	151 (55%)	12 (6%)	4.76 (3.22 – 7.14)
Coronary angioplasty	74 (27%)	30 (15%)	1.92 (1.28 – 2.94)
Coronary artery bypass grafting	66 (24%)	30 (15%)	1.75 (1.15 – 2.70)

* See text for eligibility criteria

• A history of

 Smoking

 Previous MI

 Hypertension

• Impaired left ventricular function based on echocardiography result or angiography

• Severity of AMI using the number of vessels affected (vessels were considered affected if a lesion of 50% or more was noted in the angiography report)

• Demographic factors: age, sex, ethnicity, socioeconomic status and Carstairs socio-economic deprivation category derived from postcode of residence

Baseline patient characteristics were tabulated. Categorical variables were tested for statistical significance using the χ^2 ^test. Continuous variables were tested using the Student t test comparing patients seen by cardiologists and those seen by other physicians.

The relative odds of drug and procedure use by speciality of physician were calculated. Specific variables included the use of thrombolytic therapy, β blockers, aspirin, ACE inhibitors among clinically appropriate groups, and the use of exercise testing, coronary angiography, angioplasty and coronary artery bypass grafting (CABG).

Cox proportional hazards models were used to investigate the effect on survival of seeing a cardiologist. Multivariate models were fitted to control for the effect of patient and hospital characteristics. Controlled factors included: age, sex, comorbidity, hospital, distance from patients residence to hospital and Carstairs socio-economic deprivation category in the first instance. The effect of angiography, revascularisation and the use of effective medicines (aspirin and/or β blockers and/or thrombolysis – eligibility criteria listed below) were subsequently introduced into the model to investigate whether these affected outcome. The factors were included in the model either because they are known to confound the association between type of physician and survival or are known to be associated with survival and were not comparable between the two groups.

Eligibility criteria for appropriate use of effective medicines [13].

Thrombolytic therapy

• No warfarin therapy on admission

• ST-segment elevation on initial ECG

• Less than 12 hours since onset of chest pain

• Systolic BP less than 180 mmHg and diastolic BP less than 110 mmHg at presentation

• No severe CVA, gastrointestinal disease or chronic liver disease

Aspirin

• No haemorrhagic complication

• No severe CVA, gastrointestinal disease or chronic liver disease or renal failure

• No warfarin therapy on admission

β blockers

• No chronic lung disease

• No cardiogenic shock, hypotension, complete heart block or decompensated heart failure

**Figure 1:**
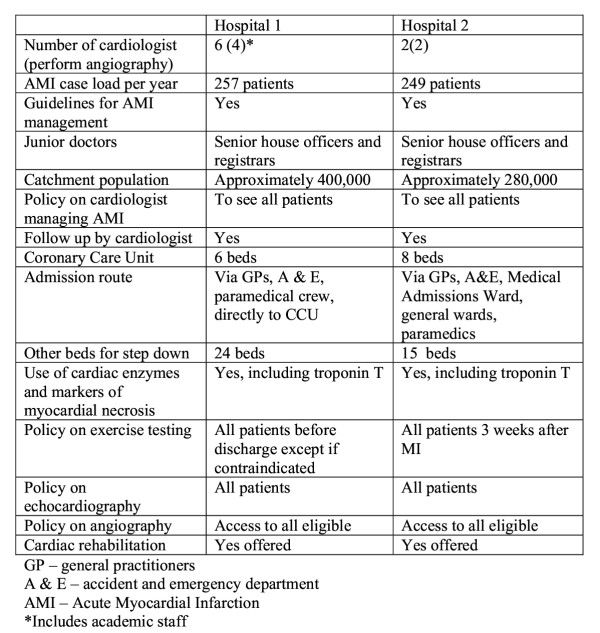
Characteristics of study hospitals.

The characteristics of the two hospitals are summarised figure 1. The results from the two hospitals were combined in order to make our findings more generalisable. The general characteristics did not show important differences in the organisation of care between the hospitals. It is unlikely there are important unmeasurable factors associated with a particular hospital that might cause confounding, but hospital of care is still included as a variable in the multivariate analysis to control for centre effect. Patients from the two hospitals undergo procedures in the same specialist cardiothoracic hospital.

The main outcome was eighteen-month survival defined as time between date of AMI and death.

Statistical analysis was carried out using Stata (Version 6.0).

In general for Cox regression analysis, the number of events i.e. deaths (and similarly the number of non-events) per variable modelled should be at least 10 [12].

Ethical approval was obtained from the two local research ethics committees of the hospitals involved in the study. Permission from the director of research and Caldicott guardians of data in each hospital was also obtained.

## Results

Medical records were obtained for 94% of eligible patients (476 of 506 patients). Four patients did not have enzyme changes of myocardial infarction or were clear cases of miscoding. The mean age of the cohort was 69.3 years (SD 13.2) and 34.5% were female. The patients with missing records did not differ from those with records in terms of age or sex using information from routine data (mean age was 70.4 years with SD 11.1; 36.2% were female). There were no significant differences in the proportion of data missing between the two hospitals including those for eligibility to effective medications and co morbidities.

### General characteristics

Patients in our sample seen by a cardiologist were younger, with a higher proportion of males to females. They were more likely to have a history of CHD prior to myocardial infarction, but less likely to have a history of congestive heart failure or hypertension (Table 1). Patients with impaired mobility or who smoked were less likely to be seen by a cardiologist. The difference in the proportion of patients with echocardiographic evidence of impaired left ventricular function complicating their myocardial infarction by physician seen was not significant.

### Drug and procedure use

The likelihood of receiving effective medication (thrombolytic therapy, β blockers, aspirin) was higher among patients seen by a cardiologist compared with those not seen by a cardiologist. Furthermore, patients seen by a cardiologist were more likely to have undergone exercise testing, angiography and revascularisation procedures (Table 2). The use of ACE inhibitors was not more frequent among cardiologists.

### Survival

Univariate analysis indicated that patients seen by a cardiologist had better survival (hazard ratio 0.16, 95% CI 0.10 to 0.25).

### Effect of seeing a cardiologist after controlling for patient characteristics

The survival of patients seen by a cardiologist was still better than that for those not seen by a cardiologist (hazard ratio 0.22, 95% CI 0.14 to 0.25) after controlling for the confounding effect of pre-existing comorbidity, age, sex, hospital first seen, deprivation and distance (Table 3). The effect of seeing a cardiologist remained after excluding patients that died within a week of MI. The hazard ratio was 0.21 (95% CI 0.12 – 0.37).

**Table 3:** Cox regression analysis for eighteen month survival comparing cardiologists and non-cardiologist physicians and controlling for potential confounders.

Variable		Mortality (%)	Adjusted hazard ratio (CI) *	Adjusted hazard ratio (CI) #
Seen by a Cardiologist	Yes	30 (11%)	0.22 (0.14 – 0.38)	0.70 (0.33 – 1.46)
	No	88 (44%)		
Comorbidity	Yes	15 (25%)	1.11 (0.62 – 1.99)	0.98 (0.49 – 1.98)
	No	107 (26%)		
Hospital	A	58 (24%)	1.01 (0.67 – 1.51)	1.08 (0.71 – 1.73)
	B	65 (28%)		
Age (years)	30 – 40	1 (8%)	1.05 (1.03 – 1.08)	1.03 (0.99 – 1.06)
	41 – 60	8 (7%)		
	61 – 70	18 (17%)		
	71 – 99	96 (40%)		
Sex	Males	64 (21%)	0.91 (0.62 – 1.35)	0.83 (0.44 – 1.55)
	Females	58 (34%)		
Distance			1.0 (0.97 – 1.04)	0.96 (0.88 – 1.03)
Angiography	Yes	12 (7%)	-	0.87 (0.24 – 3.17)
	No	112 (36%)		
Revascularisation	Yes	14 (13%)	-	0.25 (0.04 – 1.52)
	No	110 (30%)		
Effective medicines^γ^	Yes	48 (14%)	-	0.15 (0.07 – 0.29)
	No	76 (56%)		

* Adjusted for patient characteristics, hospital # Adjusted for patient characteristics, hospital and the use of effective medicines and procedures γ *Access to aspirin, and/or β blockers and/ or thrombolysis if appropriate*

### Effect of seeing a cardiologist after controlling for patient characteristics and use of effective intervention

The effect of seeing a cardiologist on survival became non-significant once access to angiography, revascularisation procedures and effective medicines (aspirin and/or β blockers and/or thrombolysis) had been adjusted for. The hazard ratio was 0.70 (95% CI 0.33 – 1.46). The fully adjusted model indicated that the most important factor affecting survival was access to effective medication. (Table 3).

## Discussion

This study improves our understanding of the care of AMI by cardiologists and general physicians in UK hospitals. Access to a cardiologist was univariately associated with better survival. This effect remained after controlling for the effect of patient characteristics, including the presence of comorbidity, but disappeared when the confounding effect of access to effective medicines was controlled for. As noted in the analysis, patients seen by a cardiologist were more likely to have been prescribed these medicines and to have had exercise testing, angiography and revascularisation. This implies that the survival advantage associated with being seen by a cardiologist is due to the more frequent use of effective medicines and is similar to findings by Chen J et al in the United States [3].

Previous studies have shown a high level of miscoding in routine data for AMI patients (RM Norris cited in [2]). The use of a case note review improved the findings of this study by assisting in ascertaining the diagnosis of myocardial infarction and ensuring the accuracy and completeness of the data used in the analysis. The possibility remains that some cases not seen through the cardiology department and not recorded in the patient administration system were missed. This is likely to be a small number and unlikely to invalidate the findings. Identifying all patients with infarction is a major shortcoming of many studies that estimate mortality among patients seen by cardiologists [2]. This study includes all patients irrespective of where they were managed in the hospital; however patients dying from myocardial infarction before arriving at the hospital were not included.

All cases diagnosed by the managing clinician as AMI with ECG changes and found to have enzyme changes indicating myocardial necrosis were included in the analysis after the case notes were reviewed. This may have involved some misclassification due to misdiagnosis, but this is likely to occur at random, affecting only a small number of cases, and can only underestimate the effect of each factor.

Limitations of this study include potential for bias in the allocation of patients to treatments and physicians (ie cardiologist/non cardiologist) that were not measured. Another possible limitation is bias arising from medical case notes which were unobtainable. Records from routine data were used to examine whether those with missing records differed in any systematic way from those with available records; it was found that they did not differ in age or sex. The study did not use any criteria for judging clinical appropriateness of the procedures used. Incomplete recording of information in the case notes hampered the determination of appropriateness for effective medication. However there was no difference in the proportion of missing data between the two hospitals.

Since doctors may selectively be referring younger patients with lower comorbidity to a cardiologist, we controlled for age and comorbidity in the analysis. However, residual confounding from age and comorbidity could still account for some of the observed difference in survival.

All patients admitted to the Coronary Care Unit (CCU) in one of the hospitals are seen by a cardiologist and have a higher chance of accessing thrombolysis, angiography and revascularisation. A cardiologist also sees the majority of patients admitted to the CCU in the second hospital. The protocol for the management of AMI in both hospitals stipulates a cardiology review of all AMI patients. Access to a cardiologist may be a proxy measure of access to effective treatment and it may not be the trigger for effective treatment. These limitations mean that the findings should be interpreted with a degree of caution. In addition our inability to use formal appropriateness criteria limits the interpretation of findings.

Despite these limitations the results indicate that, in the short term, acute trusts can improve survival of patients by increasing the use of effective medicines among general physicians.

## Conclusions

We observed better survival among patients seen by a cardiologist compared with patients with no access to a cardiologist, among a cohort of patients already admitted with AMI. This effect was entirely explained by the more frequent use of effective medicines by cardiologists in the multivariate analysis. About eight hospitals in the UK Royal College of Physicians survey have no cardiologist and another 30% have a single cardiologist [2]. It will take time to provide the minimum of two cardiologists per hospital as recommended in the CHD NSF. There are several reasons why a hospital may benefit from appointing a cardiologist, ranging from the treatment of a specific subgroup of patients that will benefit from revascularisation to prompt management of angina patients via rapid access chest clinics. However, in the short term hospitals can improve the survival of patients admitted with AMI by improving access to effective medicines. Coordination of care between cardiologists and general physicians and targeted interventions using feedback from audit, research and peer education are likely to lead to more frequent use of effective cardiovascular medicines by general physicians.

## Competing interest

None declared

## Authors' contributions

All authors contributed in writing the paper, in addition, IA collected the data and conducted the analysis, DK conceived the idea, BA provided advice on statistical analysis, JP provided input in the conduct of the study, PW contributed in refining the research question and discussing the findings of paper.

## Pre-publication history

The pre-publication history for this paper can be accessed here:


